# Tetraspanin Tspan9 regulates platelet collagen receptor GPVI lateral diffusion and activation

**DOI:** 10.1080/09537104.2016.1254175

**Published:** 2016-12-29

**Authors:** Elizabeth J. Haining, Alexandra L. Matthews, Peter J. Noy, Hanna M. Romanska, Helen J. Harris, Jeremy Pike, Martina Morowski, Rebecca L. Gavin, Jing Yang, Pierre-Emmanuel Milhiet, Fedor Berditchevski, Bernhard Nieswandt, Natalie S. Poulter, Steve P. Watson, Michael G. Tomlinson

**Affiliations:** ^a^ School of Biosciences, College of Life and Environmental Sciences, University of Birmingham, Birmingham, UK; ^b^ Department of Pathology, Medical University of Łódź, Poland; ^c^ PSIBS Doctoral Training Centre, School of Chemistry, University of Birmingham, Birmingham, UK; ^d^ Department of Experimental Biomedicine, University Hospital, University of Würzburg, Würzburg, Germany; ^e^ INSERM U1054, CNRS, UMR 5048, Centre de Biochimie Structurale, Montpellier University, France; ^f^ Institute of Cancer and Genomic Sciences, College of Medical and Dental Sciences, University of Birmingham, Birmingham, UK; ^g^ Institute of Cardiovascular Sciences, College of Medical and Dental Sciences, University of Birmingham, Birmingham, UK

**Keywords:** GPVI, platelet, single particle analysis, super-resolution imaging, tetraspanin, Tspan9

## Abstract

The tetraspanins are a superfamily of four-transmembrane proteins, which regulate the trafficking, lateral diffusion and clustering of the transmembrane proteins with which they interact. We have previously shown that tetraspanin Tspan9 is expressed on platelets. Here we have characterised gene-trap mice lacking Tspan9. The mice were viable with normal platelet numbers and size. Tspan9-deficient platelets were specifically defective in aggregation and secretion induced by the platelet collagen receptor GPVI, despite normal surface GPVI expression levels. A GPVI activation defect was suggested by partially impaired GPVI-induced protein tyrosine phosphorylation. In mechanistic experiments, Tspan9 and GPVI co-immunoprecipitated and co-localised, but super-resolution imaging revealed no defects in collagen-induced GPVI clustering on Tspan9-deficient platelets. However, single particle tracking using total internal reflection fluorescence microscopy showed that GPVI lateral diffusion was reduced by approximately 50% in the absence of Tspan9. Therefore, Tspan9 plays a fine-tuning role in platelet activation by regulating GPVI membrane dynamics.

## Introduction

Platelets are small, anucleate cells that are essential for haemostasis by aggregating to form a thrombus at sites of vascular damage. However, platelets also cause thrombosis by aggregating to block blood flow in diseased arteries following the rupture of collagen-rich atherosclerotic plaques, which can lead to heart attack and stroke. Platelet GPVI is established as the major platelet-activating collagen receptor [1–4], but also binds laminin [5] and fibrin [6, 7]. GPVI has only a minor role in haemostasis, but is important in arterial thrombosis, ischaemic stroke and maintaining vascular integrity during inflammation [8, 9]. The GPVI signalling pathway is well characterised and is initiated by phosphorylation of immunoreceptor tyrosine-based activation motifs (ITAMs) in the FcRγ chain dimer that is associated with GPVI [1, 4]. This is mediated by Src family tyrosine kinases and results in the recruitment of the tyrosine kinase Syk to the ITAMs. This is followed by downstream phosphorylation and assembly of a signalosome nucleated by the adapter protein LAT, phospholipase Cγ2 (PLCγ2) activation, Ca^2+^ mobilisation, platelet shape change, granule secretion, integrin activation and platelet spreading and aggregation [1, 4]. GPVI recognises collagen as a dimer, and a proportion of GPVI exists as a dimer on resting platelets, with dimer levels increasing upon platelet activation [10, 11]. GPVI has been reported to be lipid raft-associated [12–14] and more recently to be a component of a distinct type of microdomain formed by tetraspanin transmembrane proteins [15]. However, it is currently unknown how GPVI lateral mobility in the plasma membrane and compartmentalisation into membrane microdomains regulates its activation.

The tetraspanins are a superfamily of 33 four-transmembrane proteins in humans which interact with specific “partner proteins” and regulate their biosynthesis, intracellular trafficking, lateral diffusion in the plasma membrane and clustering [16, 17]. Tetraspanins can self-associate and appear to form nanoclusters spanning approximately 100 nm and containing approximately ten molecules of a single type of tetraspanin [18]. To date five tetraspanins have been investigated on platelets using knockout mice: CD9, CD63, CD82, CD151 and Tspan32. Mice deficient in either CD151 or Tspan32 have similar phenotypes, namely a partial defect in platelet aggregation and haemostasis that is proposed to be due to impaired outside-in signalling of the major platelet integrin αIIbβ3 [19–21]. Conversely, deficiency in either CD9 or CD63 causes a mildly hyperactive platelet phenotype without affecting haemostasis [22, 23]. CD82 deficiency results in more substantially hyperactive platelets, with enhanced haemostasis in vivo and enhanced clot retraction in vitro [24]. Platelets express additional tetraspanins, but their functions on platelets are yet to be characterised [25–27].

We previously identified Tspan9 as a tetraspanin that is expressed on platelets, but not several other cell types tested [15]. In the current study, we report the first characterisation of a Tspan9-deficient mouse. The mice were viable and had normal platelet count, size and levels of major platelet antigens. However, we demonstrate that Tspan9 contributes to GPVI-induced platelet activation and regulates its membrane dynamics.

## Methods

### Mice

Tspan9 knockout mice were generated from C57BL/6 mouse embryonic stem cells containing a gene-trap vector (clone IST11668F5) at the Texas Institute for Genomic Medicine, Houston, Texas, USA. Heterozygous breeding pairs were used to generate Tspan9 knockout mice with litter-matched controls, and mice were aged at least 8 weeks before use in experiments. GPVI-deficient mice [28] were kindly provided by Prof Jerry Ware (University of Arkansas for Medical Sciences, Little Rock, Arkansas, USA). Mouse experiments at the Biomedical Services Unit, University of Birmingham, UK, were performed in accordance with the Animals (Scientific Procedures) Act 1986. Mouse experiments in Würzburg, Germany, were approved by the District Government of Lower Franconia (Bezirksregierung Unterfranken).

### Antibodies and reagents

The rabbit anti-Tspan9 polyclonal antibody was as previously described [15], anti-α-tubulin monoclonal DM1A was from Sigma, anti-phosphotyrosine monoclonal 4G10 was from Millipore, anti-LAT phospho-specific antibodies were from Abcam, anti-human GPVI monoclonal HY101 for biochemical experiments was kindly provided by Dr Peter Smethurst (NHS Blood and Transplant, Cambridge, UK), anti-human GPVI monoclonal 1G5 for microscopy was kindly provided by Dr Elizabeth Gardiner (The John Curtin School of Medical Research, Canberra, Australia) [29] and mouse IgG1 isotype control monoclonal MOPC-21 was from Sigma. Fluorescently labelled monoclonal antibodies used for flow cytometry and single particle tracking were from Emfret, apart from anti-mouse integrin α6 (AbD Serotec), anti-mouse CLEC-2 17D9 [30] and anti-ADAM10 and anti-CD9 (R&D Systems). Atto 647N and 565 fluorescent dyes were from Sigma, and 3,3′-dihexyloxacarbocyanine iodide (DiOC6) membrane dye was from Molecular Probes. Platelet agonists were cross-linked collagen-related peptide (CRP) from Prof Richard Farndale (Cambridge University, UK), Horm collagen from Takeda, ADP from Sigma, protease-activated receptor (PAR)-4 peptide (AYPGKF) from Alta Bioscience and arachidonic acid from Cambridge Bioscience.

### Flow cytometry

Mouse platelet flow cytometry was performed on whole blood, and data were collected using CellQuest and a FACSCalibur (BD Biosciences).

### Immunohistochemistry

Paraffin sections of 5 μm from formalin-fixed blocks, of wild-type and Tspan9-deficient mouse tissues, were processed for immunohistochemistry as described previously [31].

### Platelet preparation

Human and mouse platelet-rich plasma and washed platelets were isolated from whole blood as previously described [32, 33]. Consent for human blood was obtained from each donor, and platelet preparation was carried out with ethical approval. Mouse whole blood counts were assessed using an ABX Pentra 60 haematology analyser (Block Scientific).

### Platelet biochemistry

For platelet stimulation and subsequent detection of protein tyrosine phosphorylation, platelets were pre-treated with 10 µM lotrafiban, 2 units/mL apyrase and 10 µM indomethacin, and then stimulated and subjected to lysis and western blotting as described [33]. For co-immunoprecipitation experiments, 1 × 10^9^ washed human platelets were lysed in 1% Brij97 lysis buffer [15] and pre-cleared with 20 µL protein G sepharose beads for 60 minutes at 4°C. Pre-cleared lysates were immunoprecipitated with isotype control (MOPC-21) or anti-human GPVI (HY101) antibodies, coupled to protein G sepharose, by rotating for 90 minutes at 4°C. Samples were washed in 1% Brij97 lysis buffer, separated by sodium dodecyl sulphate polyacrylamide gel electrophoresis (SDS-PAGE) and western blotted for Tspan9 and GPVI. Western blots were probed with IRDye fluorescent secondary antibodies (LI-COR Biosciences) and imaged and quantified using the Odyssey Infrared Imaging System (LI-COR Biosciences), or were probed with horseradish peroxidase-conjugated secondary antibodies and developed with enhanced chemiluminescence (ECL) and film.

### In vitro platelet function assays

Lumi-aggregometry was conducted as previously described [34]. For the platelet flow adhesion assay, flow cells in a 24-well plate format (Fluxion) were coated with 30 µg/mL collagen for one hour and blocked with 5 mg/mL of denatured, filtered fatty acid-free bovine serum albumin at 4 °C overnight. Blood was incubated with 0.2 µM DiOC6 membrane dye at 37°C for 10 minutes and then perfused through the flow cell at a shear rate of 1000 s^−1^ at 37°C using the BioFlux 200 Microfluidic Flow System (Fluxion). Images were continuously taken by fluorescence microscopy using a TE2000 Nikon microscopy system, with a 40× plan APO/1.4 NA oil-immersion DIC objective.

### In vivo platelet function assays

Bleeding time was assessed in the mice using a previously described tail bleeding assay [34]. Intravital assessment of in vivo thrombus formation, after topical application of FeCl_3_ to mesenteric or carotid arteries, was done as previously described [35].

### Platelet spreading and staining for microscopy

Mouse or human platelet-rich plasma was diluted to 2 × 10^7^ platelets/mL in modified Tyrode’s buffer [33]. Mouse platelets were pre-incubated with 5 μg/mL of rat anti-mouse GPVI clone JAQ3 [36] for 10 minutes at 37°C and then allowed to spread on glass-bottom dishes coated with 10 µg/mL collagen for 45 minutes at 37°C. Human platelets were pre-incubated with 5 μg/mL of mouse anti-human GPVI clone 1G5 and then similarly spread on collagen. Adhered platelets were washed once with phosphate-buffered saline (PBS) and fixed with 10% formalin solution for 10 minutes. Fixed platelets were rinsed with PBS three times and permeabilised with 0.1% Triton X-100 in PBS for 5 minutes. Fixed platelets were then washed three times with PBS. Mouse platelets were stained with an Alexa 647-conjugated goat anti-rat antibody and Alexa 488-conjugated phalloidin in blocking buffer (1% bovine serum albumin and 2% goat serum in PBS). Human platelets were co-stained with 5 μg/mL mouse anti-human GPVI clone 1G5 and 2 μg/mL rabbit anti-Tspan9, washed three times with PBS and stained with Alexa 647-conjugated goat anti-mouse antibody and Alexa 488-conjugated goat anti-rabbit antibody in blocking buffer.

### Confocal reflection and fluorescence microscopy

Fixed and stained platelet samples were imaged by confocal microscopy using the 488 nm Argon laser line and 633 nm laser line (He/Ne laser) of a Leica SP2 inverted confocal with the 63 × 1.4 NA oil objective. Reflected light was collected from the 488 nm excitation wavelength. Confocal z-stacks of platelets were acquired using the Leica TSC SP2 software with images taken at 0.25 µm intervals. Four fields of view were taken per treatment, per experiment. To determine the extent of co-localisation between Tspan9 and GPVI on human platelets spread on collagen, the two-colour image was first de-noised in ImageJ using an image restoration algorithm based on the assumption of Poisson noise [37]. Background subtraction was performed in MATLAB using a rolling balling approach in which the radius of the ball was selected to be greater than image features (1 µm). A Costes’ approach was used to automate threshold selection for signal isolation and to determine P values for each z-stack [38], where the null hypothesis was randomly distributed signal within the region of interest. This region of interest was defined by manual segmentation of the platelets and collagen fibres using the interference reflectance channel. The Manders’ coefficients (M1 and M2) were used as the co-localisation measure [39].

### Direct stochastic optical reconstruction microscopy imaging and cluster analysis

Fixed and stained mouse platelet samples were imaged in direct stochastic optical reconstruction microscopy (dSTORM) mode using a 100 × 1.49 NA total internal reflection fluorescence (TIRF) objective lens on a Nikon N-STORM system consisting of a Ti-E stand with Perfect Focus, Agilent MLC400 high-power laser bed (647 nm laser line) and Andor iXon Ultra 897 EMCCD camera. Samples were imaged in a PBS buffer containing catalase (1 μg/mL), mercaptoethylamine-HCl (100  mM), glucose (40% v/v) and glucose oxidase (50 μg/mL) to induce fluorophore blinking. Twenty thousand frames were captured using Nikon NIS Elements AR v4.5, with an exposure time of 9.2 ms, gain 300 and conversion gain 3, and then reconstructed using STORM analysis module 3.2. Detected molecules (fluorescent blinks) of a photon count of less than 500 were filtered out of the final reconstruction. Samples were drift corrected and rendered using Gaussian rendering. Cluster analysis was performed on the dSTORM data in MATLAB, using a custom-made algorithm based on Ripley’s K-function [40] with modifications as described [41]. For the analysis, 2 × 2 µm regions of interest were analysed for clustering with the spatial scale r = 50 nm used to generate the pseudo-coloured cluster heat map, where red indicates high degrees of clustering. Binary maps were generated from these cluster heat maps by threshold application. Areas of the heat map that were above the upper threshold of L(50) = 100 were defined as clusters. Information on the clusters was extracted from the binary map, including the number of molecules and the size of clusters. A total of 179 regions of interest from wild-type and 177 regions of interest from Tspan9-deficient mice, from four independent experiments, were analysed for GPVI clustering on collagen.

### GPVI and CD9 single particle tracking

Fab fragments from anti-GPVI monoclonal antibody JAQ1 [42] and anti-CD9 monoclonal antibody Nyn.H3 were coupled to Atto 647N or 565 as described previously [43]; 500 µL of washed mouse platelets at 2 × 10^7^/mL was allowed to spread directly on cleaned glass coverslips for 35 minutes at 37°C. Non-adhered platelets were removed by gentle washing with modified Tyrode’s buffer [33]. The relevant antibodies and the membrane dye DiOC6 were incubated for 10 minutes at 37°C with the spread platelets at a final concentration of 10 ng/mL for JAQ1, 5 ng/mL for CD9, 50 ng/mL for IgG controls and 0.2 µM for DiOC6. Unbound antibody was removed by gentle washing with Tyrode’s buffer. Platelets were imaged at 37°C using an Olympus IX81-ZDC dual TIRF microscope with a Hamamatsu EM-CCD digital camera using an Olympus 150× UAPO N 1.45 NA immersion oil TIRF objective with Violet Diode 400–405 nm, ArgonIon 457–514 nm and Green Diode 561 nm lasers. Five hundred images were captured every 130 ms to generate a video. Laser power was kept to a minimum, to avoid untimely bleaching of the fluorophores, and camera gain was set to maximum. At least ten different fields of view were imaged for each condition. Single particle tracking videos were analysed using PaTrack software and the previously described parameters for trajectory inclusion criteria and determination of motional modes (Brownian, confined or directed) [44].

## Results

### Generation of Tspan9-deficient mice

**Figure 1.**
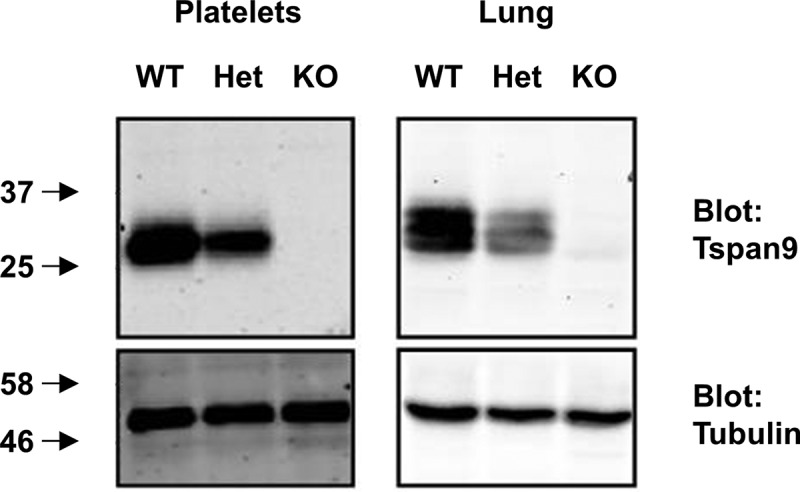
Tspan9 is not detectable in platelets and lung from Tspan9-deficient mice. Platelets from wild-type (WT), heterozygote (Het) and Tspan9-deficient (KO) mice were lysed in 1% Triton X-100 lysis buffer, the proteins separated by SDS-PAGE and western blotted for Tspan9 (upper panel) and α-tubulin to confirm equal protein loading (lower panel), using the Odyssey Infrared Imaging System. Mouse lung samples were homogenised in 1% Triton X-100 lysis buffer and western blotted as described for the platelets.

We previously generated Tspan9 antibodies to demonstrate expression of this tetraspanin by platelets and in certain tissues such as lung [15]. In order to investigate the function of Tspan9, a Tspan9-deficient mouse was generated using gene-trap embryonic stem cells on the C57BL/6 background. The mice were viable, normal in weight, born at expected Mendelian frequencies when bred as heterozygotes and fertile as homozygotes (data not shown). The platelets were normal in size and count, as were other blood cell parameters (Table I). Loss of Tspan9 protein expression was confirmed by western blotting of lysates from platelets and lung, which showed an approximately 50% reduction of protein in heterozygotes and complete loss in homozygote knockouts (Figure 1).

**Table I.** Tspan9-deficient mice have platelets of normal count and size, and normal numbers of other blood cell parameters. Whole blood from wild-type (WT) and Tspan9-deficient (KO) mice was analysed using an ABX Pentra 60 haematology analyser from Block Scientific. Data are presented as means and standard deviations from between 18 and 20 litter-matched pairs of mice. No significant differences were revealed using unpaired t-tests.

Blood parameter	WT	KO
Platelet count (10^3^/mm^3^)	892 ± 159	924 ± 281
Platelet volume (µm^3^)	5.36 ± 0.25	5.35 ± 0.27
Plateletcrit (%)	0.29 ± 0.07	0.29 ± 0.07
White blood cell count (10^3^/mm^3^)	8.24 ± 3.94	8.30 ± 4.04
Red blood cell count (10^6^/mm^3^)	10.3 ± 1.12	10.1 ± 0.73
Haemoglobin concentration (g/dL)	9.15 ± 0.61	9.34 ± 0.64
Haematocrit (%)	29.4 ± 2.4	30.7 ± 5.1
Red blood cell distribution width (%)	12.8 ± 0.78	12.8 ± 0.54
Mean corpuscular volume (µm^3^)	47.9 ± 1.3	48.3 ± 1.3
Mean corpuscular haemoglobin (pg)	15.1 ± 0.51	16.9 ± 7.9
White cell percentages:		
- Lymphocytes	86.2 ± 6.9	85.1 ± 7.4
- Monocytes	4.18 ± 1.97	4.90 ± 2.48
- Neutrophils	7.69 ± 4.24	8.29 ± 4.35
- Eosinophils	1.17 ± 1.41	0.97 ± 1.37
- Basophils	0.76 ± 0.90	0.65 ± 1.02

### Tspan9 expression in mouse tissues

**Figure 2.**
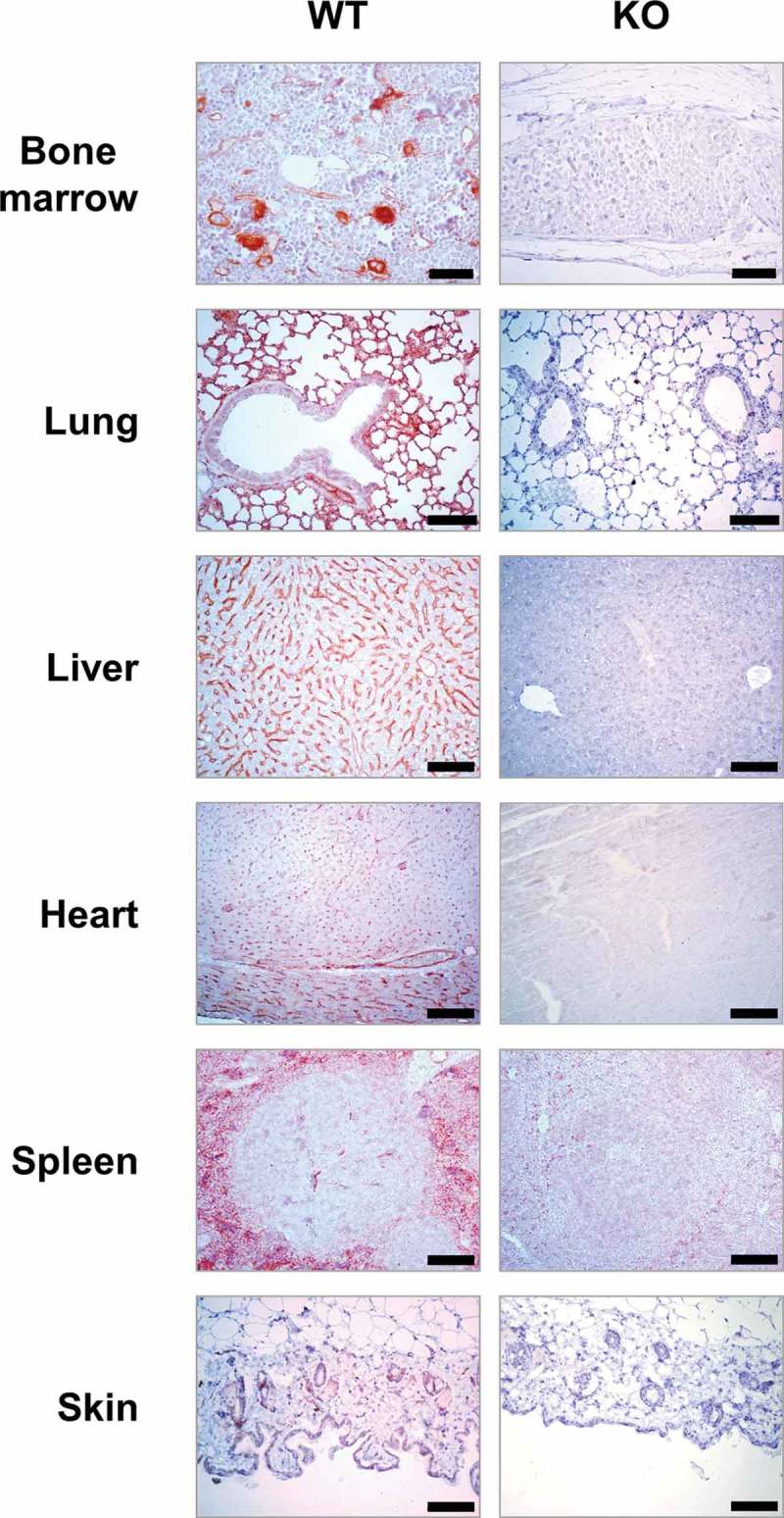
Tspan9 expression in mouse tissues is restricted to megakaryocytes and a limited range of other cell types including some endothelial and epithelial cells. Immunohistochemistry was performed on sections from wild-type (WT) and Tspan9-deficient tissues as a negative control (KO). The scale bar is 100 µm.

To further investigate the Tspan9 expression profile, expression in various mouse tissues was assessed by immunohistochemistry, using tissues from Tspan9-deficient mice as a negative control. Analyses of bone marrow revealed Tspan9 expression in relatively large cells characteristic of megakaryocytes (Figure 2), which was consistent with our previous identification of Tspan9 expression in platelets and their megakaryocyte progenitors [15]. Blood vessels also appeared positive for Tspan9 (Figure 2). Tspan9 was prominent in lung, again in agreement with our previous western blotting data [15], and appeared to be localised to the endothelial cells of interalveolar walls and the basal aspect of bronchial epithelial cells (Figure 2). Tspan9 was also expressed in the sinusoidal endothelial cells of the liver, small vessels and capillaries of the heart, the red pulp of the spleen, which is rich in sinusoidal venous endothelial cells, and cells of the glandular layer of the skin (Figure 2). In each case, no Tspan9 expression was detected in Tspan9-deficient tissues (Figure 2). These data are consistent with Tspan9 expression in the platelet/megakaryocyte lineage, in addition to a limited range of other cell types including some endothelial and epithelial cells.

### Tspan9-deficient platelets have a mild but specific GPVI activation defect

To determine whether platelets were defective in levels of major surface glycoproteins in the absence of Tspan9, they were first analysed by flow cytometry. Expression of the following was not affected by the lack of Tspan9: the platelet-activating receptors GPVI and CLEC-2, the tetraspanin CD9, the metalloproteinase ADAM10, the von Willebrand factor receptor GPIb and the integrins αIIbβ3, α2β1 and α6β1 (Table II).

**Table II.** Tspan9-deficient platelets express normal levels of the major platelet surface glycoproteins. Whole blood from wild-type (WT) and Tspan9-deficient (KO) mice were stained with fluorescent-conjugated antibodies, and platelet-specific staining was determined following gating by size. Isotype negative control staining was subtracted, and geometric mean fluorescence intensities with standard deviations are presented from between 3 and 8 pairs of litter-matched mice. No significant differences were determined using unpaired t-tests.

Surface protein	WT	KO
α2 integrin	18.0 ± 0.9	16.2 ± 0.6
αIIb integrin	1113 ± 108	1153 ± 73
α6 integrin	158 ± 4.1	161 ± 0.9
ADAM10	33.7 ± 1.7	38.2 ± 2.4
CD9	905 ± 65	883 ± 61
GPIb	504 ± 11	489 ± 25
GPVI	125 ± 4.5	120 ± 3.3
CLEC-2	109 ± 26	110 ± 26

**Figure 3.**
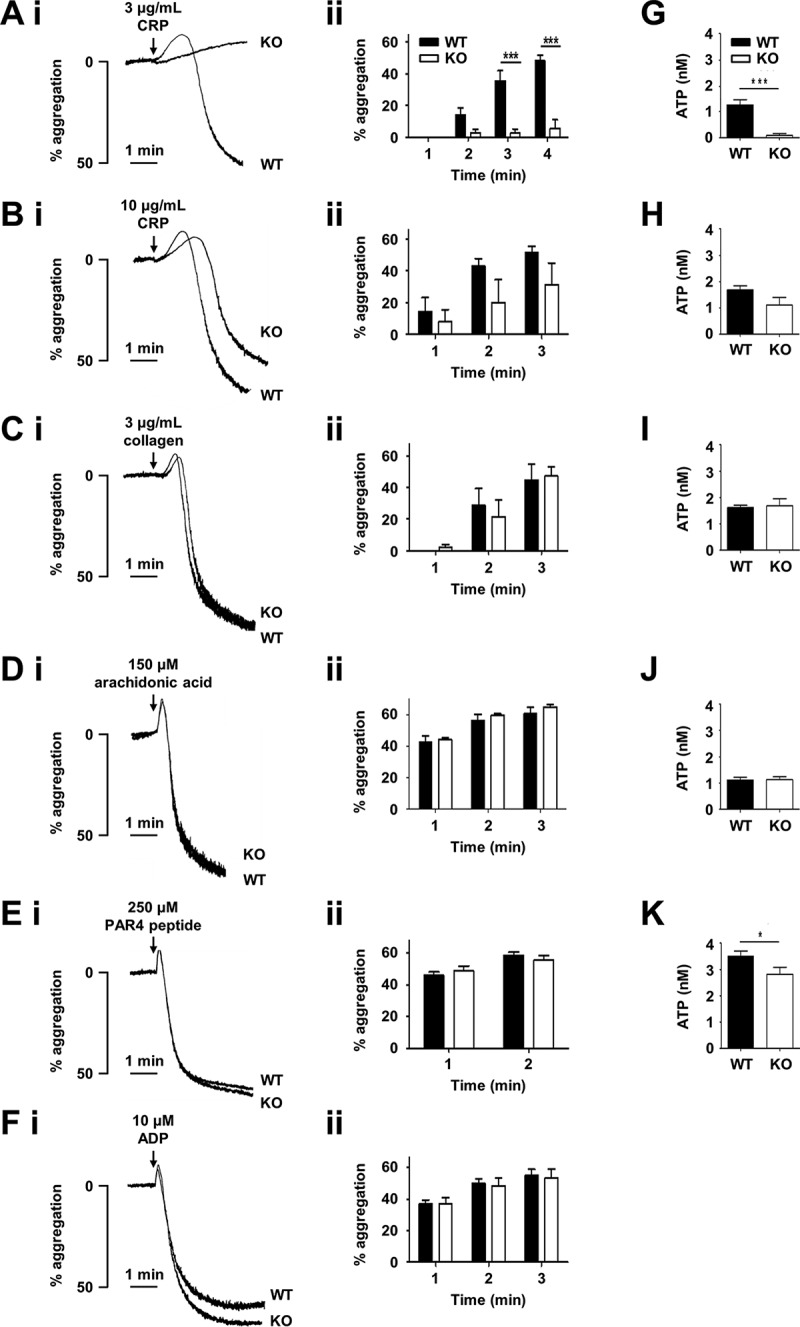
Tspan9-deficient platelets have a mild but specific defect in GPVI-induced platelet aggregation and secretion. Wild-type (WT) and Tspan9-deficient (KO) platelets in platelet-rich plasma were activated with the GPVI-specific agonist cross-linked collagen-related peptide (CRP) at [A, G] 3 µg/mL and [B, H] 10 µg/mL, [C, I] collagen, [D, J] arachidonic acid, [E, K] PAR4 peptide and [F] ADP. [A–F] Aggregation was measured by light transmission with stirring. [i] Representative traces and [ii] quantitated percentage aggregation each minute are shown. A two-way ANOVA was performed on arcsine-transformed data followed by a Bonferroni post-test (*** denotes P < 0.001). [G–K] ATP secretion was measured by lumi-aggregometery. Significance was determined by an unpaired t-test (* denotes P < 0.05 and *** denotes P < 0.001). Error bars represent the standard error of the mean from between three and nine pairs of litter-matched mice.

**Figure 4.**
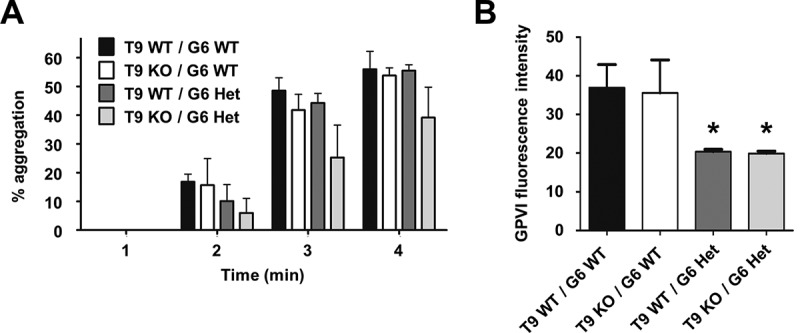
Tspan9-deficient platelets have normal aggregation responses to collagen. [A] Tspan9 (T9)-deficient mice were crossed with GPVI (G6)-deficient mice to generate Tspan9-deficient mice on a GPVI heterozygote (Het) or wild-type (WT) background. Platelet-rich plasma from these mice was activated with 1 µg/mL collagen, and aggregation was measured by light transmission with stirring. Quantitated percentage aggregation data for each minute are shown; error bars represent standard error of the mean from seven mice per genotype. A two-way ANOVA performed on arcsine-transformed data revealed no significant differences. [B] Whole blood was analysed by flow cytometry for GPVI, as described in the legend for Table II. Error bars represent standard deviation from three to four mice per genotype. Significance was determined by a one-way ANOVA followed by a Tukey’s post-test (* denotes P < 0.05).

To assess platelet function, aggregation assays were performed using platelet-rich plasma. Tspan9-deficient platelets were defective in aggregation induced by the GPVI-specific agonist cross-linked collagen-related peptide (CRP) at an intermediate concentration of 3 µg/ml (Figure 3A). This was a mild defect which was overcome at 10 µg/ml of CRP (Figure 3B). No defect was evident with 3 µg/ml of the physiological GPVI agonist collagen (Figure 3C), which also binds to integrin α2β1. The aggregation defect was specific to GPVI because no defect was observed in response to arachidonic acid, PAR4 peptide or ADP (Figure 3D–F), which activate platelets via distinct G protein-coupled receptors (GPCRs). Similarly, no defect was observed at lower concentrations of collagen or the GPCR agonists (data not shown). Consistent with these aggregation data, a lack of dense granule secretion was evident when platelet-rich plasma was stimulated with 3 µg/ml of CRP (Figure 3G), but not in response to 10 µg/ml CRP (Figure 3H), collagen (Figure 3I) and arachidonic acid (Figure 3J). A significant defect in PAR4 peptide-induced secretion was observed, but this was only a 20% decrease (Figure 3K); since there was no PAR4 aggregation defect (Figure 3E), a role for Tspan9 in PAR4-induced platelet activation appears unlikely. To test whether a Tspan9 collagen aggregation phenotype could be revealed at a 50% reduced surface expression level of GPVI, which could make a role for Tspan9 in regulating GPVI more critical, Tspan9-deficient mice were crossed with GPVI-deficient mice [28]. This allowed the effect of Tspan9 deficiency to be investigated on a GPVI-heterozygous background. However, no significant defect in collagen-induced aggregation was detected in the absence of Tspan9, even at the relatively low collagen concentration of 1 µg/ml (Figure 4A). Approximately 50% reduced surface levels of GPVI in the heterozygous genotypes were confirmed by flow cytometry (Figure 4B).

**Figure 5.**
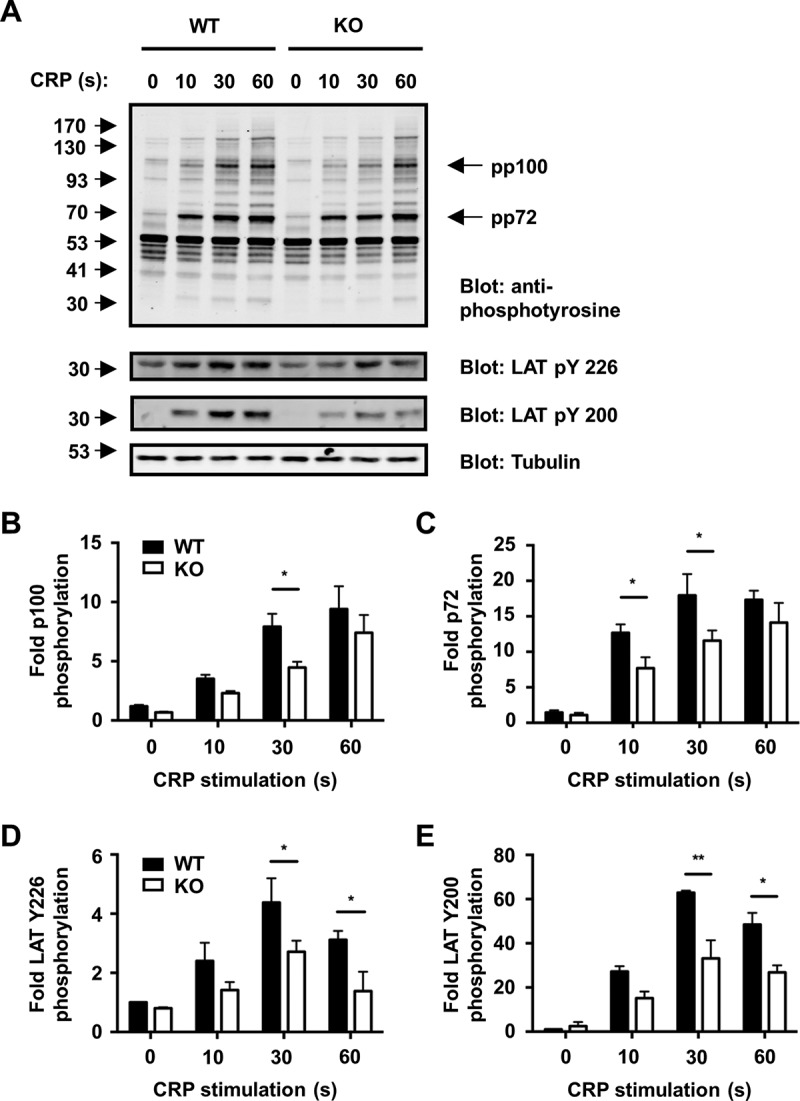
GPVI signalling is impaired in Tspan9-deficient platelets. [A] Washed platelets from wild-type (WT) and Tspan9-deficient (KO) mice were activated with 3 μg/mL cross-linked collagen-related peptide (CRP), and samples were taken at 0, 10, 30 and 60 second time points and lysed in 1% Triton X-100 lysis buffer. Proteins were separated by SDS-PAGE and blotted with the 4G10 anti-phosphotyrosine antibody (upper panel) or phospho-specific antibodies to LAT phosphotyrosines 226 or 200 or α-tubulin to control for protein loading (lower panels). The blots were visualised using the Odyssey Infrared Imaging System, and band intensities of the whole cell phosphoproteins of [B] 100 kD (pp100) and [C] 72 kD (pp72), LAT phosphotyrosine [D] at amino acid position 226 and [E] 200 were quantitated and normalised to tubulin. Significance was determined by a two-way ANOVA followed by a Bonferroni post-test (* denotes P < 0.05 and ** denotes P < 0.01). Error bars represent the standard error of the mean from three pairs of litter-matched mice.

To test whether Tspan9-deficient platelets were defective in GPVI-induced signalling, tyrosine phosphorylation was measured by western blotting of whole cell lysates following stimulation with CRP. A mild reduction in intensity of some phosphoproteins was apparent in the absence of Tspan9 over the 60 second time course (Figure 5A), including the phosphoproteins of 100 and 72 kD (Figure 5B and C). Another affected phosphoprotein was the transmembrane adapter protein LAT, which exhibited a significant 40%–50% reduction in phosphorylation on tyrosines at positions 200 and 226 (Figure 5D and E).

Together these data demonstrate that Tspan9-deficient platelets have mild defects in GPVI-induced tyrosine phosphorylation and platelet aggregation and secretion, despite normal surface expression of GPVI.

### Tspan9 is not essential for haemostasis or thrombosis

**Figure 6.**
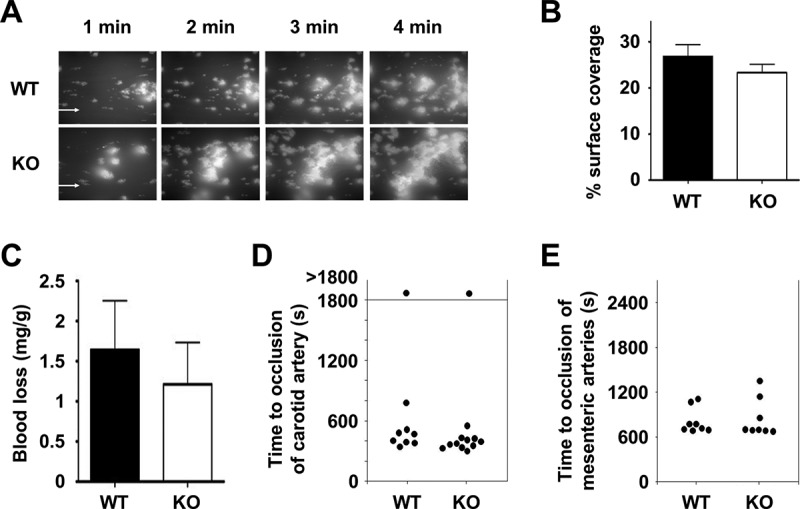
Tspan9-deficiency does not affect adhesion to collagen under flow, haemostasis or arterial thrombosis. [A] To assess adhesion to collagen under flow, heparinised whole blood from wild-type (WT) and Tspan9-deficient (KO) mice was incubated with 0.2 µM DiOC6 membrane dye and perfused over a collagen-coated flow cell (30 µg/mL) using the Fluxion Bioflux system at a shear rate of 1000 s^−1^. Representative fluorescence images are shown after 1, 2, 3 and 4 minutes, with flow from left to right. [B] Coverage of the flow cell by aggregates was measured using thresholding in ImageJ. Quantitation revealed no significant differences following a two-way ANOVA with Bonferroni post-test on arcsine-transformed data. Error bars represent standard error of the mean from three litter-matched pairs of mice. [C] To assess haemostasis, wild-type (WT) and Tspan9-deficient (KO) mice were assessed for tail bleeding by excising 2 mm tail tips from anaesthetised mice and measuring the weight of blood lost as a factor of mouse weight. No significant difference was determined using an unpaired t-test. Error bars represent standard error of the mean from 20 litter-matched pairs of mice. [D-E] To assess chemical injury-induced thrombosis, platelets were fluorescently labelled with Dylight-488-conjugated anti-GPIX antibody. Mice were anaesthetised, and the carotid artery [D] or mesentery [E] was exteriorised. FeCl_3_-induced chemical injury was induced via topical application to the vessels. Time to complete occlusion of the vessel was measured using fluorescence intravital microscopy. No significant difference was determined using an unpaired t-test for panels D or E.

A series of experiments were designed to test whether Tspan9-deficient platelets had any defect in aggregate formation on collagen under in vitro flow conditions, and whether the Tspan9-deficient mice had any haemostasis or thrombosis defects. No defect in aggregate formation on collagen under in vitro flow conditions was observed in the absence of Tspan9 (Figure 6A and B), and there was no haemostasis phenotype using the tail bleed assay (Figure 6C). Furthermore, in a ferric chloride injury model of in vivo thrombosis, the absence of Tspan9 had no effect on vessel occlusion times in large (carotid) or intermediately sized (mesenteric) arteries (Figure 6D and E).

### Tspan9 associates with GPVI

**Figure 7.**
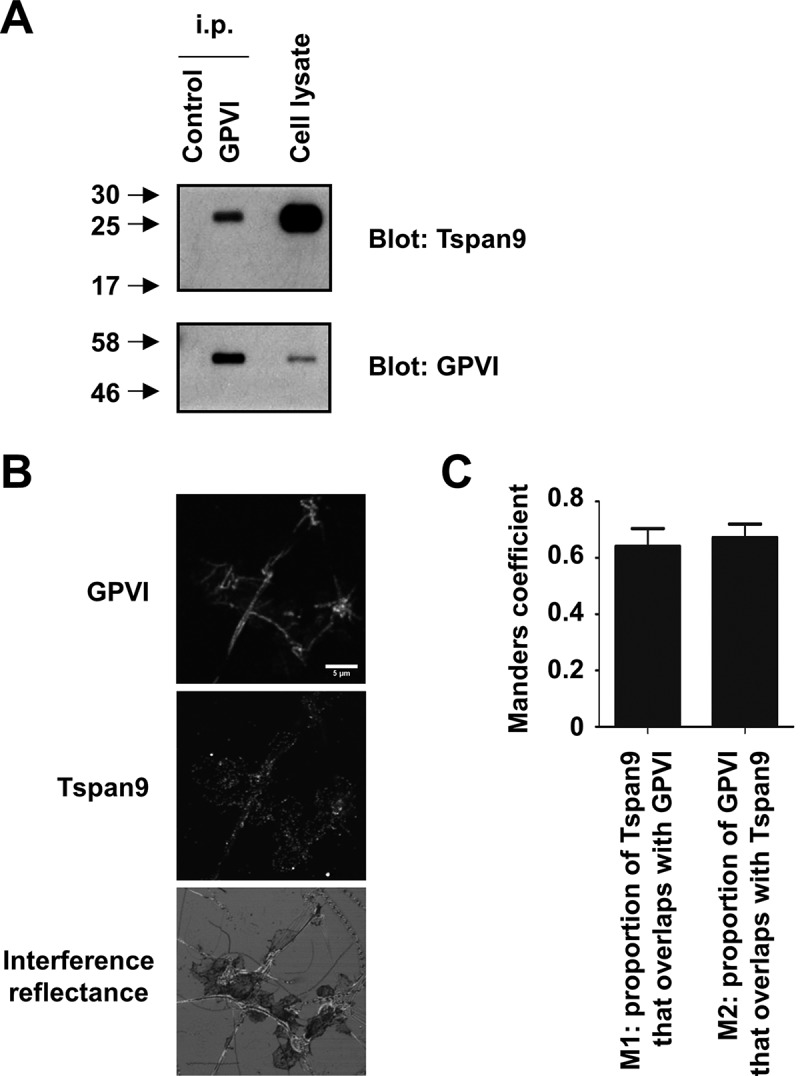
Tspan9 associates with GPVI on human platelets. [A] Washed human platelets were lysed in 1% Brij97 lysis buffer and subjected to immunoprecipitation with isotype control or anti-GPVI antibodies. Immunoprecipitates were analysed by SDS-PAGE and western blotting for Tspan9 (top panel) and GPVI (lower panel) using ECL and film. [B] Human platelets were spread on collagen and imaged by confocal microscopy following immunostaining for GPVI (top panel) and Tspan9 (middle panel). Interference reflectance was used to determine the position of the platelets and collagen fibres (lower panel). [C] The Manders’ coefficients (M1 and M2) were calculated from the confocal stacks to quantify the degree of overlap. All Costes P values for individual stacks were <0.001, indicating significant partial co-localisation in all cases.

To elucidate the mechanism by which the loss of Tspan9 affects GPVI activation, we first investigated whether GPVI interacts with Tspan9. Human platelets were lysed in 1% Brij97 and then subjected to GPVI immunoprecipitation and Tspan9 western blotting. This demonstrated that Tspan9 was co-immunoprecipitated by GPVI (Figure 7A). However, the interaction was not observed in more stringent 1% digitonin lysis buffer (data not shown), suggesting a weak or indirect interaction. To test whether GPVI and Tspan9 co-localise, co-immunofluorescence confocal microscopy was carried out on human platelets spread on collagen. GPVI co-localised with collagen fibres and partially with Tspan9 (Figure 7B). Quantitation of these data using the Manders’ coefficients revealed that approximately 60% of Tspan9 staining overlapped with GPVI, and vice versa (Figure 7C), which is consistent with the similar copy numbers of Tspan9 and GPVI on platelets [15]. These two sets of experiments suggest that a proportion of Tspan9 associates with GPVI.

### Tspan9 does not affect GPVI clustering on platelets spread on collagen

**Figure 8.**
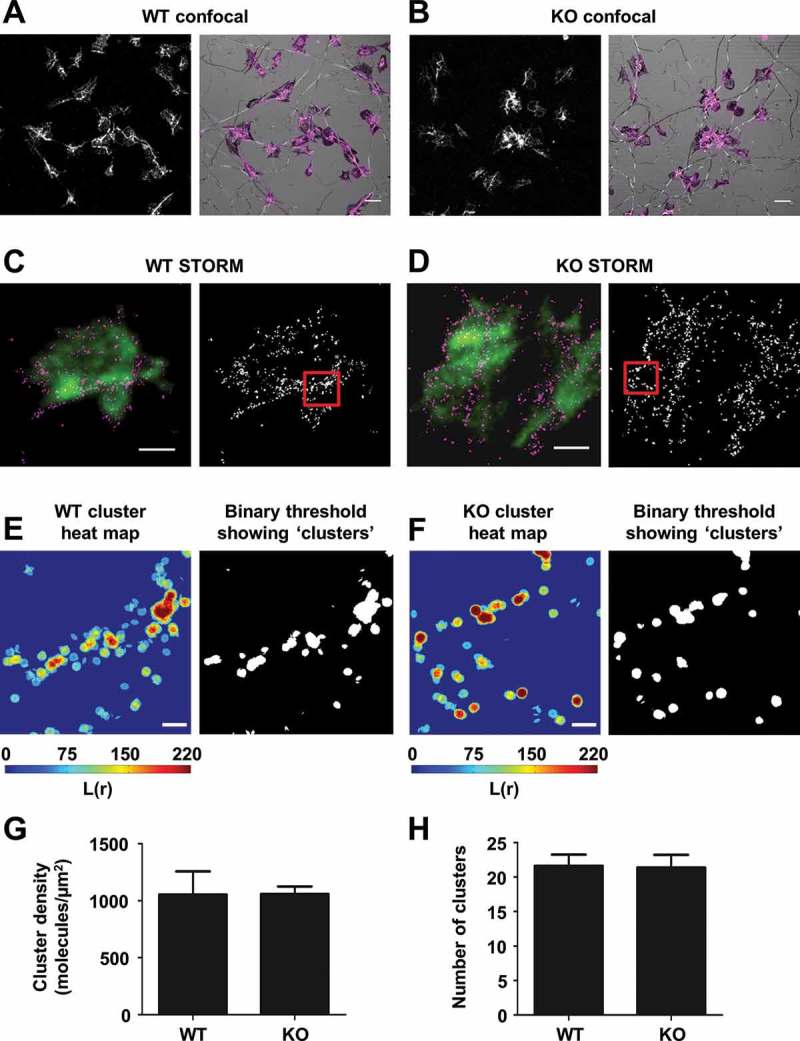
Tspan9 does not regulate GPVI clustering on collagen. [A] Wild-type (WT) and [B] Tspan9-deficient (KO) mouse platelets were spread on collagen for 45 minutes, immunostained for GPVI with JAQ3 Fab and anti-rat-Alexa 488, and imaged using confocal microscopy (left panels). Collagen fibres and platelets were imaged by confocal reflection and GPVI overlaid in magenta (right panels). [C] Wild-type (WT) and [D] Tspan9-deficient (KO) mouse platelets, labelled for GPVI (JAQ3 and anti-rat-Alexa 647), were imaged using super-resolution dSTORM TIRF (magenta; left panels), which is overlaid onto a TIRF image of the F-actin labelled with phalloidin-Alexa 488 (green). For quantitative cluster mapping analysis of GPVI localisation, 2 × 2 µm regions of interest were taken inside platelets and along collagen fibres (right panels). Heat maps of the clusters in the regions of interest from [E] wild-type and [F] Tspan9-deficient platelets are shown (left panels). Binary images of the clusters, following thresholding of the heat map, show clustering of GPVI along the collagen fibres (right panels). Quantification of GPVI clustering on collagen fibres was made using a custom-made algorithm in MATLAB, and presented as [G] cluster density and [H] number of clusters that contain at least three molecules per cluster. Data represent means of medians, and the error bars are standard errors of the mean from four independent experiments. In total, 179 regions were analysed for wild-type mice and 177 regions for Tspan9-deficient mice. Scale bars are 5  μm for panels A and B, 2  μm for panels C and D, and 0.25 μm for panels E and F.

It is well established that certain tetraspanins can promote the clustering of their partner proteins [16]. To determine whether GPVI clustering on collagen was affected in the absence of Tspan9, GPVI was imaged by confocal microscopy using Fab fragments of GPVI monoclonal antibody JAQ3, which does not interfere with collagen binding [36], on human platelets spread for 45 minutes on collagen. GPVI co-localised with collagen fibres on both wild-type and Tspan9-deficient platelets, but no differences were apparent at this resolution (Figure 8A and B). To investigate GPVI clustering at the super-resolution level, GPVI was imaged using dSTORM (Figure 8C and D). Cluster analysis (Figure 8E and F) showed no difference in GPVI density (Figure 8G) or number of GPVI clusters (Figure 8H), suggesting that Tspan9 does not affect GPVI clustering on collagen. However, this assay was performed on fully spread platelets, which may not detect delays in GPVI clustering.

### Tspan9 promotes GPVI lateral diffusion

**Figure 9.**
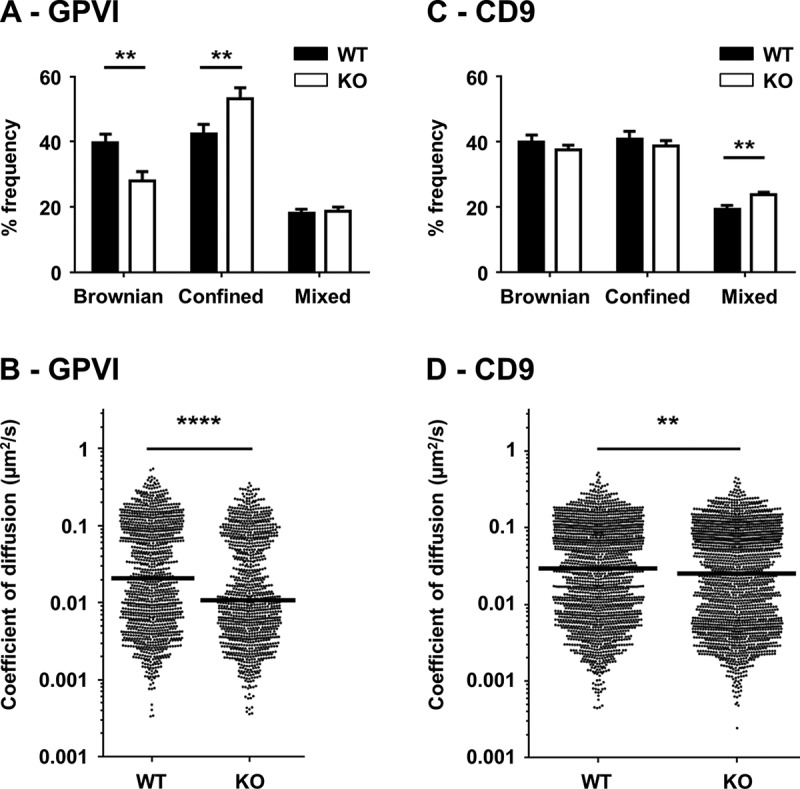
Tspan9 promotes GPVI lateral mobility in the plasma membrane. [A] Platelets from wild-type (WT) and Tspan9-deficient (KO) mice were allowed to spread on glass coverslips and stained with fluorescent-conjugated anti-GPVI Fab fragments. For each of at least ten different fields of view, 500 images were captured every 130 ms to generate single particle tracking videos, which were analysed using PaTrack software and the motion type (Brownian, confined or directed) determined using previously described parameters [43]. Error bars represent the standard error of the mean from six pairs of litter-matched mice, and significance was determined by one-way ANOVA followed by Sidak’s multiple comparison test (** denotes P<0.01 and **** denotes P<0.0001). [B] The coefficient of diffusion was extracted from the trajectory mean-squared displacement over time and presented as a scatter plot in which each point corresponds to a distinct GPVI particle. The median value is indicated by a horizontal line. Statistical significance was determined by Kruskal–Wallis followed by a Dunn’s multiple comparison test for diffusion coefficients. [C and D] The experiments were performed as described for panels A and B, except that platelets were stained with fluorescent-conjugated anti-CD9 Fab fragments.

Tetraspanins are emerging as regulators of the lateral diffusion of the transmembrane proteins with which they interact [45–48]. To determine whether Tspan9 regulates GPVI lateral mobility, single particle tracking was developed for the first time in platelets. Mouse platelets were allowed to spread on glass coverslips and then incubated with single Atto 647N-labelled anti-GPVI Fab fragments. The plane of the plasma membrane was then imaged using TIRF microscopy every 130 ms for a total of 500 times. The trajectory type and apparent diffusion coefficient of individual labelled particles were then determined using PaTrack software [43]. In wild-type platelets, three different motion types were detected for GPVI trajectories: purely Brownian motion (40% of total trajectories), confined or restricted diffusion (42%) and trajectories with both Brownian and confined elements (18%) (Figure 9A). In Tspan9-deficient platelets, the three motion types were also displayed by GPVI molecules, but the percentage of Brownian motion significantly decreased by 30% compared to wild type, and confined or restricted trajectories significantly increased by 26% (Figure 9A). This significantly reduced the median apparent diffusion coefficient of the GPVI population by 43%, from 0.021 on wild-type to 0.012 µm^2^/s on Tspan9-deficient platelets (Figure 9B).

To determine whether the absence of Tspan9 could similarly affect the diffusion of other membrane proteins, platelets were co-labelled with single Atto 565-labelled CD9 Fab. In contrast to GPVI, there was no significant difference in the relative frequency of confined or restricted CD9 trajectories in wild-type versus Tspan9-deficient platelets, although there was a small but significant 4.5% increase in the relative frequency of CD9 particles that displayed mixed (Brownian and confined) diffusion (Figure 9C). This change in diffusion behaviour affected the total population of tracked CD9 particles, causing a small but significant difference in the median apparent diffusion coefficient from 0.029 on wild-type to 0.025 µm^2^/s on Tspan9-deficient platelets, representing a 14% decrease (Figure 9D).

In summary, the GPVI lateral diffusion and signalling defects in the absence of Tspan9 suggest that this tetraspanin maintains a relatively mobile population of GPVI molecules, with the potential to rapidly cluster and signal upon ligand engagement.

## Discussion

This study represents the first characterisation of the Tspan9-deficient mouse. We have discovered that Tspan9 is necessary for normal platelet protein tyrosine phosphorylation, aggregation and secretion following activation through the collagen receptor GPVI. Moreover, GPVI lateral mobility on the cell surface was reduced by approximately 50% in the absence of Tspan9. Since GPVI clustering is important for its activation and signalling [10, 11], a reduction in its lateral diffusion could explain the observed platelet aggregation and secretion defects. No defects in GPVI clustering on collagen were observed by super-resolution imaging, but these experiments were done on fully spread platelets and would not detect a delay in the kinetics of clustering.

Tetraspanins are thought to function by interacting with specific partner proteins and regulating their trafficking and membrane dynamics. Definitive tetraspanin/partner pairs include tetraspanin CD151 with laminin-binding integrins [17], CD81 with the B-cell co-receptor CD19 [49] and the TspanC8 subgroup of tetraspanins with a disintegrin and metalloproteinase 10 (ADAM10) [26]. Their interactions can be detected by co-immunoprecipitation in relatively stringent lysis conditions (e.g. 1% Triton X-100 or digitonin). Moreover, defects in partner protein trafficking to the cell surface and/or membrane dynamics can be observed following tetraspanin knockdown or knockout, and tetraspanin deficiency typically phenocopies partner protein deficiency [16]. Tspan9 and GPVI have similar expression levels in platelets [15], but the data in the present study are not definitive in showing that GPVI is a bona fide partner for Tspan9. We found that GPVI and Tspan9 could be co-immunoprecipitated from human platelets, but only in relatively non-stringent 1% Brij lysis buffer, and the interaction was not maintained in 1% digitonin (data not shown). In addition, only a partial co-localisation was observed by confocal microscopy. GPVI surface expression levels were not affected by the loss of Tspan9, suggesting that it does not regulate steady-state expression of GPVI on the cell surface, but GPVI lateral mobility was reduced. Finally, Tspan9 deficiency only weakly phenocopied GPVI deficiency, since the GPVI-specific platelet activation and aggregation defects were not detected in response to the physiological agonist collagen, even at low agonist concentration and in GPVI-heterozygous platelets in which a Tspan9 function might have been more critical. Moreover, the aggregation and secretion defects in response to the GPVI-specific agonist CRP were overcome by increased CRP concentrations. It is possible that signalling induced by the collagen-binding integrin α2β1 [50, 51] is sufficient to overcome the mild GPVI-induced aggregation defect. In vivo Tspan9-deficient mice had no arterial thrombosis defects in GPVI-dependent models. It is therefore possible that Tspan9 does not directly interact with GPVI, but modulates the collagen receptor by regulation of another protein that is important for GPVI membrane dynamics. Alternatively, Tspan9 could directly engage in relatively low affinity interactions with a subpopulation of GPVI molecules, enhancing their lateral diffusion by an undefined mechanism.

Two previous single particle tracking studies have reported the promotion of partner protein lateral diffusion by a tetraspanin. Firstly, epidermal growth factor receptor lateral diffusion is reduced by almost 50% following knockdown of tetraspanin CD82 in the HeLa epithelial cancer cell line [45]. Secondly, ADAM10 lateral diffusion is almost twofold increased by Tspan15 expression in the U2OS osteocarcinoma cell line [46]. In contrast, two other single particle tracking studies have shown that tetraspanins confine their partners. The proportion of confined α6 integrin is decreased by 75% on a mammary cell line upon CD151 knockdown, consistent with the idea that CD151 clusters the integrin within nanodomains [48]. Furthermore, CD19 lateral diffusion is increased by 2.5-fold on B cells from CD81-knockout mice [47]. It is proposed that CD81 confines CD19 into nanoclusters that can interact with activated B-cell receptors to promote signalling [47]. The mechanisms by which tetraspanins exert their effects on partner protein dynamics are currently unknown.

CD9 lateral diffusion was also reduced in the absence of Tspan9, but this was a weaker effect than for GPVI. Moreover, unlike GPVI, no decrease in CD9 Brownian motion and increase in confined motion types were observed. The lateral diffusion of CD9 on wild-type platelets (0.029 µm^2^/s) was substantially lower than previously reported for CD9 on PC3 and HeLa epithelial cancer cells (0.23 and 0.24 µm^2^/s, respectively) [43, 52], and for the related CD81 on HepG2 liver cancer cells (0.11 µm^2^/s on non-polarised and 0.07 µm^2^/s on polarised cells) [53]. This may be due to the relatively high density of platelet CD9, as the second most highly expressed surface protein on these cells [15, 25]. Indeed, the relatively high proportion of confined CD9 on platelets is consistent with the capacity for CD9 to form platforms in which individual CD9 molecules can be transiently confined [43].

In addition to expression in platelets, immunohistochemistry demonstrated Tspan9 expression in endothelial cells of the lung, liver, heart and spleen, and some lung epithelial cells. However, no effects of Tspan9 deficiency were observed in the gross morphology of these organs. Elucidation of tetraspanin function using knockout mice can be complicated by functional redundancy between tetraspanins, which share relatively strong protein sequence identity. For example, CD9 and CD81 share 45% identity, and double-deficient mice have macrophage dysfunction leading to a phenotype that resembles chronic obstructive pulmonary disease; neither single knockout has detectable disease [54]. Tspan9 has relatively strong sequence identity with Tspan4 (55%) and CD53 (43%), for which knockout mice phenotypes have yet to be reported. CD53 is restricted to leukocytes [55] and is absent from platelets [56, 57], so its expression does not appear to overlap with Tspan9. Tspan4 appears to be more widely expressed [58] but not by platelets [56, 57]. These expression profiles could explain how a platelet phenotype has been identified in the Tspan9-deficient mice. Moreover, co-expression of Tspan9 and Tspan4 in endothelial and epithelial cells could explain the lack of a noticeable Tspan9 phenotype in these cells.

Tspan9 has recently been identified as a novel host factor for the infection of the U2OS osteocarcinoma cell line with alphaviruses [59]. These are small enveloped plus-sense RNA viruses which can cause arthritis and encephalitis following transmission by mosquitos. There are presently no treatments or vaccine for alphaviruses. Tspan9 is localised to the plasma membrane and early and late endosomes in these cells. It is necessary on early endosomes for viral membrane fusion with the endosomal membrane, which allows infection by releasing the viral nucleocapsid into the cytoplasm [60]. The Tspan9-deficient mice may be a useful model system to assess the importance of Tspan9 in alphavirus infection in vivo.

In summary, we have performed the first characterisation of Tspan9-deficient mice and identified a mild but specific defect in platelet activation via the collagen receptor GPVI. Single particle tracking demonstrated altered diffusion characteristics of GPVI in the absence of Tspan9. We propose that Tspan9 maintains a relatively mobile population of GPVI molecules, which facilitate the rapid assembly of GPVI clusters on exposed collagen following injury.
